# A Macaque Model to Study Vaginal HSV-2/Immunodeficiency Virus Co-Infection and the Impact of HSV-2 on Microbicide Efficacy

**DOI:** 10.1371/journal.pone.0008060

**Published:** 2009-11-30

**Authors:** Federica Crostarosa, Meropi Aravantinou, Onome J. Akpogheneta, Edith Jasny, Andrew Shaw, Jessica Kenney, Michael Piatak, Jeffrey D. Lifson, Aaron Teitelbaum, Lieyu Hu, Anne Chudolij, Thomas M. Zydowsky, James Blanchard, Agegnehu Gettie, Melissa Robbiani

**Affiliations:** 1 Center for Biomedical Research, Population Council, New York, New York, United States of America; 2 AIDS and Cancer Virus Program, SAIC-Frederick, Inc., National Cancer Institute at Frederick, Frederick, Maryland, United States of America; 3 Tulane National Primate Research Center, Tulane University Health Sciences Center, Covington, Louisiana, United States of America; 4 Aaron Diamond AIDS Research Center, Rockefeller University, New York, New York, United States of America; Institut Pasteur Korea, Republic of Korea

## Abstract

**Background:**

Herpes simplex virus type-2 (HSV-2) infection enhances the transmission and acquisition of human immunodeficiency virus (HIV). This occurs in symptomatic and asymptomatic stages of HSV-2 infection, suggesting that obvious herpetic lesions are not required to increase HIV spread. An animal model to investigate the underlying causes of the synergistic action of the two viruses and where preventative strategies can be tested under such complex physiological conditions is currently unavailable.

**Methodology/Principal Findings:**

We set out to establish a rhesus macaque model in which HSV-2 infection increases the susceptibility to vaginal infection with a model immunodeficiency virus (simian-human immunodeficiency virus, SHIV-RT), and to more stringently test promising microbicides. HSV-2 exposure significantly increased the frequency of vaginal SHIV-RT infection (n = 6). Although cervical lesions were detected in only ∼10% of the animals, long term HSV-2 DNA shedding was detected (in 50% of animals followed for 2 years). Vaginal HSV-2 exposure elicited local cytokine/chemokine (n = 12) and systemic low-level HSV-2-specific adaptive responses in all animals (n = 8), involving CD4^+^ and CD8^+^ HSV-specific T cells (n = 5). Local cytokine/chemokine responses were lower in co-infected animals, while simian immunodeficiency virus (SIV)-specific adaptive responses were comparable in naïve and HSV-2-infected animals (n = 6). Despite the increased frequency of SHIV-RT infection, a new generation microbicide gel, comprised of Carraguard® and a non-nucleoside reverse transcriptase inhibitor MIV-150 (PC-817), blocked vaginal SHIV-RT infection in HSV-2-exposed animals (n = 8), just as in naïve animals.

**Conclusions/Significance:**

We established a unique HSV-2 macaque model that will likely facilitate research to define how HSV-2 increases HIV transmission, and enable more rigorous evaluation of candidate anti-viral approaches *in vivo*.

## Introduction

Herpes simplex virus type-2 (HSV-2) prevalence is between 19–50% in adults worldwide [1], [2] and increases the risk of human immunodeficiency virus (HIV) acquisition by ∼3 fold [3], [4]. HSV-2 infection is characterized by viral shedding at mucosal surfaces, occurring in the presence of lesions (clinical reactivation) or with very mild or no symptoms (subclinical reactivation). HIV acquisition risk is increased during both the symptomatic and asymptomatic phases of genital herpes. Individuals with recently acquired HSV-2 infection showed a higher incidence of HIV acquisition (22.6% per 100 person-years) compared to chronically infected people (7.5% per 100 person-years) [5]. These findings correlate with the observation that, although HSV-2 results in a chronic infection, the frequency of symptomatic genital herpes reactivation decreases with time since infection [6].

Primary and recurrent genital HSV-2 infection involves disruption of the epithelial surface. The interaction between HSV-2 and epithelial cells triggers cytokine and chemokine secretion [7] to recruit dendritic cells (DCs), macrophages, and CD4^+^ and CD8^+^ T cells to control infection and symptomatic reactivation [8]–[11]. HSV-specific CD8^+^ T cells have been documented at the dermal-epidermal junction next to peripheral sensory nerve endings in HSV-infected people, suggesting that virus reactivation occurs more frequently than first thought [8]. Reactivated shedding and lesions were shown to be rapidly cleared (within a median ∼12 h) and predominantly asymptomatic (<10% with lesions) [12]. Longer reactivation periods were associated with a higher viral loads and frequency of lesions (∼30% when shedding lasted longer than 24 h). The rapid clearance of virus is likely due to the persistence of the CD8^+^ T cells within the tissues, which are able to clear the subclinical HSV-2 reactivation [8].

HSV-2-activated CD4^+^ T cells can act as target cells for HIV, facilitating HIV acquisition. HSV-2 and HIV can also co-infect lymphocytes [13] and macrophages [14] *in vitro*, resulting in the up-regulation of HIV replication. While epithelial cells are the major targets for HSV-2 infection, human [15], [16] and macaque [17] DCs are susceptible to HSV-2 infection *in vitro* resulting in the down-modulation of their immunostimulatory functions. Notably, HSV-2-infected DCs stimulate weaker SIV-specific responses than uninfected DCs [17]. Thus, the presence of more immature DCs with suboptimal function would provide even more targets to support HIV replication in the absence of maximal immune activity. The notion that increased HIV infection can occur as a result of immune dysfunction caused by HSV-2 infection is supported by the fact that HSV-2-infected people lacking obvious lesions exhibit increased susceptibility to HIV [18], [19]. Additionally, the observation that treating HSV-2 infection with acyclovir did not affect the incidence of HIV acquisition [20], suggests that immune damage might already have occurred, and is not reversed by anti-HSV treatment. However, there is increasing evidence for subclinical reactivation of HSV-2 (90% in women and 80% in men), which is associated with microscopic lesions that contain activated CD4^+^ T cells (reviewed in [2]). Moreover, a recent report from Zhu et al revealed that cellular infiltrates at the site of HSV-2 reactivation comprised CD4^+^ and CD8^+^ T cells, as well as myeloid and plasmacytoid DCs (MDCs and PDCs), all of which persisted for months after the lesions healed, even with daily antiviral therapy [21]. The persistence of these potential HIV targets helps explain why anti-HSV therapy did not reduce HIV acquisition. Treatment of HSV infection has been shown to reduce the levels of HIV in plasma and mucosal fluids in HSV/HIV-infected men and women [22]–[24], probably by reducing the HSV-mediated activation of CD4^+^ T cells thereby lowering HIV production.

To further understand how HSV-2 infection increases HIV spread, and to more accurately test novel anti-viral approaches, a macaque mucosal HSV-2 infection model was developed. HSV-2 exposure resulted in long term HSV-2 DNA shedding, increased frequency of vaginal SHIV-RT infection, and elicited local and systemic responses. The efficacy of a microbicide under the physiological complexity of a co-infection vs SHIV-RT challenge alone was also demonstrated. Such a model is of fundamental importance to better understand the interplay of HSV-2 and HIV, and provides a useful means of testing the efficacy of preventative and therapeutic strategies.

## Methods

### Animals and Treatments

Adult female Chinese rhesus macaques (*Macaca mulatta*) were housed at the Tulane National Primate Research Center (TNPRC). Herpes B virus is prevalent in most macaque colonies [25], [26]. To limit the chance that prior infection with Herpes B virus might impact the susceptibility to HSV-2 infection, all animals used in these studies were prescreened for Herpes B virus infection and only Herpes B virus negative animals were used. Animals were injected with 30 mg of Depo-Provera i.m. and 5 weeks later intra-vaginally (i.vag.) challenged with 2×10^8^ plaque forming units (pfu) HSV-2 strain G (American Type Culture Collection, ATCC, Manassas, VA) in 1 ml of serum-free RPMI 1640 (Cellgro/Mediatech, Manassas, VA). Animals were Depo-Provera treated again (1.5–7 months after HSV-2 challenge), before being co-challenged i.vag. with 2×10^8^ pfu HSV-2 and 200 or 10^3^ TCID_50_ SHIV-RT (genomic backbone SIV_mac239_ with the RT gene of HIV-1) [27] in 1 ml of serum-free RPMI 1640. An additional 4 animals were challenged with HSV-2 intra-rectally (i.r.) before being Depo-Provera-treated (∼8–11 months after the primary HSV-2 challenge) and i.vag. co-challenged as above. A schematic describing the various challenge groups is provided in Figure S1. Control groups included SHIV-RT challenged animals receiving a single Depo-Provera treatment or a double Depo-Provera treatment (given within 3 months of each other in the absence of HSV-2). In later studies, 3 ml of the microbicide candidate gel (or placebo) was applied 30 minutes or 24 hours prior to application of 1 ml of virus [28]. Animals remained in a supine position for 20 minutes post-challenge, to allow absorption of virus. All animals and their respective treatments are summarized in Tables 1 and 2.

**Table 1:** HSV-2 exposure increases SHIV-RT infection frequency.

HSV-2 status	SHIV-RT TCID_50_	No. of Depo-Provera Treatments#	Animal No.	SHIV-RT-specific		
				Infection	IFNγ	Ab
**Naïve**	**∼200**	1X	GF15	**−**	**−**	**−**
			GF16	**−**	**−**	**−**
			GJ28	**−**	**−**	**−**
			GJ29	**−**	**−**	**−**
			GF13	**−**	**−**	**−**
	**1000**	1X	FI80	**+**	**+**	**+**
			FI81	**+**	**+**	**+**
			FI82	**+**	**+**	**+**
			DE38	**−**	**+**	**−**
			GF15	**−**	**−**	**−**
			GF16	**−**	**−**	**−/+**
			GJ43	**+**	**+**	**+**
			GJ51	**+**	**+**	**+**
			GK06	**−**	**−**	**−**
			GK05	**−**	**−**	**−**
			GJ28	**+**	**+**	**+**
			GJ29	**−**	**−**	**−**
			GF13	**−**	**−**	**−**
**Infected**	**200**	1X	GF24	**−**	**−**	**−**
			GJ82	**+**	**+**	**+**
			DT72	**+**	**+**	**+**
			CR03	**−**	**−**	**−**
			GF11^b^	**+**	**+**	**+**
			GF04^ b^	**−**	**−**	**−**
			GF10^ b^	**−**	**−**	**−**
			GF19^ b^	**−**	**−**	NA
	**1000**	1X	GF24	**+**	**+**	**+**
			GF22	**+**	**+**	**+**
			GF09	**+**	**+**	**+**
			GF10 a	**+**	**+**	NA
			GF19 a	**+**	**−**	NA
			GF04 a	**+**	**+**	NA
**Naïve**	**1000**	2X	GK06	**+**	**+**	**+**
			GK03	**+**	**−**	**+**
			GJ90	**+**	**+**	**+**
			GJ91	**+**	**+**	**+**
**Infected**	**1000**	2X	GJ42	**+**	**+**	**+**
			GJ81	**+**	**+**	**+**

#Number of Depo-Provera treatments within a 3 month window.

a indicates intra rectal (i.r.) route of HSV-2 infection.

NA, sample not available.

Note, some uninfected animals were recycled and rechallenged and, therefore are listed more than once.

**Table 2:** 30 minute pretreatment with PC-817 prevents vaginal SHIV-RT infection in HSV-2-infected macaques.

Gel	Time of gel administration before challenge	SHIV-RT TCID_50_	Animal No.	SHIV-RT-specific		
				Infection	IFNγ	Ab
**MC**	**30 min/24 h**	**1000**	GF24	**+**	**+**	**+**
			GF22	**+**	**+**	**+**
			GF09	**+**	**+**	**+**
			GJ42	**+**	**+**	**+**
			GJ81	**+**	**+**	**+**
**PC-817**	**30 min**	**1000**	GF24	**−**	**−**	**−**
			GF26	**−**	**−**	**−**
			GF28	**−**	**−**	**−**
			GF31	**−**	**−**	**−**
			GJ82	**−**	**−**	**−**
			GJ83	**−**	**−**	**−**
			GJ94	**−**	**−**	**−**
			GJ98	**−**	**−**	**−**
**PC-817**	**24 h**	**1000**	GF26	**+**	**+**	**−**
			GF28	**+**	**+**	**+**
			GJ83	**+**	**+**	**−**
			GJ94	**+**	**+**	**−**
			GJ98	**+**	**+**	**+**
**MC**	**24 h**	**200**	GF24	**−**	**−**	**−**
			GJ82	**+**	**+**	**+**
**PC-817**	**24 h**	**200**	GF26	**−**	**−**	**−**
			GF28	**−**	**−**	**−**
			GF31	**+**	**+**	**+**
			GJ83	**−**	**−**	**−**
			GJ94	**−**	**−**	**−**
			GJ98	**−**	**−**	**−**

SHIV-RT negative animals (no viral RNA in plasma and no adaptive immunity) were recycled and re-challenged as indicated.

NA, sample not available.

HSV-2/SHIV-RT and SHIV-RT-infected monkeys were treated with the mouse-human chimeric anti-CD8 mAb cM-T807 (NIH Nonhuman Primate Reagent Resource-Beth Israel Deaconess Medical Center, Boston, MA). The animals received 10 mg/ml s.c. at day 0, followed by 5 mg/kg i.v. on days 3, 7, and 10 as described in the published protocol [29].

Blood and mucosal fluid samples were collected as indicated. Vaginal and cervical pinch biopsies were collected 3–12 months post-HSV-2/SHIV-RT co-challenge. Biopsy procedures were followed with buprenorphine 0.01 mg/kg for analgesia. During all procedures monkeys were anesthetized with tiletimine/zolazepam (8 mg/kg). Blood samples for hematological analysis, immunological and virological assays were taken in the morning prior to feeding. Protocols were reviewed and approved by the Institutional Animal Care and Use Committee of TNPRC. Animal care procedures were in compliance with the regulations detailed under the Animal Welfare Act [30] and in the Guide for the Care and use of Laboratory Animals [31]. Animals that did not become infected with SHIV-RT (verified by negative plasma viremia and lack on adaptive immunity to SIV) were recycled and re-challenged as indicated.

### Virus Stocks

The generation of HSV-2 strain G stocks was performed on Vero cells (CCL-81; ATCC) [32] and virus titers were determined by plaque-forming assay [32]. Aliquots of HSV-2 (and the no virus-containing supernatant controls) were UV-inactivated as previously described [17]. Aliquots of infectious and UV-inactivated virus were stored at −80°C until use. SHIV-RT stocks were grown in PHA-activated human PBMCs (kindly provided by Disa Böttiger, Medivir AB, Sweden). Stocks were re-titered using the 174XCEM cell line (NIH AIDS Research & Reference Reagent Program) and the TCID_50_ was calculated using the Reed and Muench formula.

### Microbicide Preparation

PC-817 (Lot numbers: 100907, 101606-A, 011508, 032707-A) is 3% (w/v) Carraguard® containing 500 µM of the non nucleoside reverse transcriptase inhibitor (NNRTI) MIV-150 (Medivir AB, Sweden) [28]. 2.5% (25 mg/ml) methyl cellulose (MC) (Lot numbers: 100207, 101806-A, 032807, 011008) (Fisher, Fair Lawn, NJ) was used as the placebo gel. Gels were stored at room temperature and used within 7–10 days of preparation.

### Cell Isolation

Peripheral blood mononuclear cells (PBMCs) were isolated from EDTA blood using Ficoll-Hypaque density gradient centrifugation (Amersham Pharmacia Biotech, Uppsala, Sweden). Axillary, inguinal and mesenteric lymph nodes (LNs), as well as vaginal and cervical tissues were obtained at necropsy. Pooled LN cell suspensions were prepared as we described previously [33]. Mucosal cell suspensions were prepared by incubating the tissues with 200 µg/ml gentamycin (Invitrogen/GIBCO, Carlsbad, CA) in RPMI 1640 (Cellgro/Mediatech, Manassas, VA) 10% FBS (Cellgro/Mediatech) for 1–2 hours, and after 2 washes in PBS 1x (Cellgro/Mediatech) the tissues were cut in small pieces (approximately 2 mm) and incubated in 0.1 mg/ml Collagenase/Dispase (Roche Diagnostic Corporation, Indianapolis, IN) in RPMI 1640 (Cellgro/Mediatech) 10% FBS (Cellgro/Mediatech), containing 1 mg/ml Hyaluronidase (400–1000 U/ml) (Sigma), 0.5 mg/ml Collagenase II (Sigma) 1 mg/ml DNase I (Roche Diagnostic Corporation), at 37°C for 20 minutes, shaking. The suspension was passed through a metal sieve, the larger pieces of tissue being disrupted further using a sterile glass pestle. The tissue that was not completely disrupted was re-suspended in the medium and the procedure above was followed, for a maximum of 3 times. The final cell suspension was filtered through a 70 µm nylon cell strainer (BD-Falcon, Franklin Lakes, NJ) and washed twice with medium. Mucosal cells were stored as dry pellets for PCR.

### IFN-γ ELISPOT

The numbers of IFN-γ spot-forming cells (SFCs) in the blood and LN preparations were measured by ELISPOT [34]. Cells were cultured in triplicate (100 µl), in plates coated with anti-IFN-γ Abs (clone MD-1; Biosource International, Camarillo, CA) in the presence of UV-inactivated HSV-2 (5 pfu equivalents/cell), aldrithiol 2 (AT-2)-inactivated SIVmneE11s (300 ng/ml p27; AT-2 SIV lot # P4055), their respective no-virus microvesicle (MV) controls generated from the same cells used to generate the viral stocks (AT-2 SIV MV control lot # P3856) and 1 µg/ml ConA (Sigma) (to control for cell functionality and assay integrity). After overnight incubation at 37°C cells were lysed and the plate developed as described [35]. Spots were counted using an AID ELISPOT reader (Cell Technology, Columbia, MD). The SIV- and HSV-2-specific IFN-γ responses were calculated subtracting any background responses to the respective no-virus MV controls. Culture medium used throughout was RPMI 1640 (Cellgro/Mediatech), containing 2 mM L-glutamine (Invitrogen/GIBCO) 10 mM HEPES (N-2-hydroxyethylpiperazine-N'-2-ethanesulfonic acid) (Invitrogen/GIBCO), 50 µM 2-mercaptoethanol (Sigma) penicillin (100 U/ml) and streptomycin (100 µg/ml) (Invitrogen/GIBCO) and 1% heparinized human plasma (Innovative Research, Southfield, MI).

### Vaginal Fluid Collection

Vaginal swabs were obtained by inserting foam swabs into the vaginal cavity for 5 minutes and immersing them in 1 ml of sterile PBS containing 1% FCS (Cellgro/Mediatech) and penicillin-streptomycin. Samples were kept at 4°C prior to processing (within 24 hours). Swabs were drained of fluid and then discarded. The remaining samples were centrifuged at 3500 rpm for 10 minutes. Total fluids or aliquots of supernatants and the pelleted cells were stored at −80°C.

### Cytokine and Chemokine Analysis

Stored vaginal swab fluid was thawed and centrifuged at 3000 rpm for 10 minutes. Supernatants were then analyzed using the monkey-reactive Beadlyte human 14-plex Detection System according to the manufacturer's instructions (Upstate Biotechnology, Lake Placid, NY). The system recognizes macaque IL1β, IL2, IL3, IL4, IL5, IL6, IL12p40, CXCL8, IFNγ, TNFα, GM-CSF, CCL2, CCL3 and CCL5. Samples were analyzed using Luminex 100 or Luminex 200 (Luminex, Austin, TX) and StarStation software (Applied Cytometry Systems, Sacramento, CA).

### Flow Cytometry

A standard 4-color flow cytometry analysis was performed to examine the DCs present in macaque blood and LNs using Abs and conditions reported previously [33]. To analyze CD8^+^ T cell counts in blood of CD8^+^-depleted animals 3-color TruCount flow cytometry was used. Whole blood was stained according to the manufacturer's instruction for phycoerythrin (PE)-conjugated anti-CD8 (clone DK25; BD Pharmingen), fluorescein isothiocyanate (FITC)-conjugated anti-CD4 (clone L200; Dako), peridinin-chlorophyll-Cychrome (PerCP-Cy5.5)-conjugated anti-CD3 (clone SP34; BD Pharmingen). Samples were acquired using a FACSCalibur flow cytometer (BD Immunocytometry Systems, CA) and analyzed using FlowJo software (Tree Star, CA).

Intracellular cytokine staining (ICS) was used to detect antigen-specific T cells using minor modifications of a previously described protocol [36]. 5×10^6^ PBMCs were incubated for 6 hours at 37°C with UV-inactivated HSV-2 (5 pfu equivalents/cell), AT-2 SIV (300 ng/ml p27, lot # P4055), their respective MV controls, or 50 nM phorbol 12-myristate 13-acetate (PMA) and 1 µg/ml ionomycin (both Sigma, St. Louis, MO). The cells were stimulated in a total volume of 1 ml of RPMI 1640, containing 2 mM L-glutamine, 10 mM HEPES, 50 µM 2-mercaptoethanol, penicillin (100 U/ml) and streptomycin (100 µg/ml) and 10% heat-inactivated fetal calf serum (Cellgro/Mediatech) in 24-well plates (BD Falcon) pre-coated with anti-CD28 and anti-CD49d co-stimulatory Abs (both 10 µg/ml BD Pharmingen) cross-linked by 2.5 µg/ml GAM IgG F(ab)_2_ (KPL, Gaithersburg, MD) (for the antigen-specific responses). Brefeldin A (Sigma) was added to the cultures at 10 µg/ml for the final 4.5 hours of incubation. Stimulated cells were surface stained with PerCP-Cy5.5-conjugated anti-CD4 (clone SP34-2, BD Biosciences) and Pacific Blue-conjugated anti-CD3 Abs (clone L200, BD Biosciences), washed in FACS Wash (5% FBS and 0.1% sodium azide in PBS) and fixed overnight with 4% paraformaldehyde (Electron Microscopy Sciences). After permeabilization for 20 minutes at 4°C using FACS permeabilizing solution (BD Biosciences) cells were stained with FITC-conjugated anti-TNFα (clone MAb11, Biolegend, San Diego, CA), PE-conjugated anti-IL-2 (clone N7.48A) and APC-conjugated anti-IFNγ (clone 45-15, both Miltenyi, Auburn, CA) Abs for 1 hour at room temperature in permeabilizing solution followed by washing and fixation in FACS fixation solution (BD Biosciences). Five hundred thousand events were collected gating on lymphocytes (small, non granulated cells) on a BD LSRII (BD Biosciences) and data were analyzed using FlowJo software.

### HSV-2 DNA PCR

DNA was extracted from 200-600 µl aliquots of vaginal fluid, the pelleted material obtained from fluids, and vaginal and cervical biopsy samples using the QIAamp DNA blood Mini Kit (QIAGEN, Valencia CA, USA) according to the manufacturer's instructions and eluted into 100 µl elution buffer. DNA was extracted from vaginal and cervical biopsy tissues using ATL buffer and Proteinase K (Qiagen), followed by extraction with the QIAamp DNA blood Mini Kit as above. HSV-2 DNA shedding was determined by measuring the presence of the HSV-2 UL30 gene (which encodes the viral DNA polymerase) using a nested PCR. This assay was able to reproducibly detect HSV-2 UL30 DNA signals from 0.5 infected cells (single replicates) or 0.0005 infected cells (at least 2 positives in 6 replicates; Fig. S2) within 45,000 uninfected cells. Sequencing analysis confirmed that the 146 bp band detected was within the HSV-2 UL30 gene. The external primers used to amplify part of the HSV-2 UL30 gene were: Ext UL30 Fw (5′- GCATCATCTACGGGGACACG) and Ext UL30 Rev (5′- TCGGCGGTGAGGACAAAGTC). The internal UL30 primers used were: UL30 Fw (5′- GACACGGACTCCATTTTCGT), UL30 Rev (5′- AGCAGCTTGGTGAACGTTTT). The GAPDH gene was used as positive assay control with primer sequences: GAPDH Fw (5′- GAAGGTGAAGGTCGGAGT), GAPDH Rev (5′- GAAGATGGTGATGGGATTTC). Both external and nested PCR assays were performed in a 20 µl reaction mixture containing 0.01 µM UL30 primers or 0.02 µM GAPDH primers, 4 µM of dNTPs (Invitrogen, Carlsbad Ca, USA), 0.4units of Hotstart Taq DNA polymerase (Qiagen); the external reaction contained up to 200 ng of DNA. After the activation of the Hotstart Taq polymerase at 95°C for 12 minutes, DNA was amplified by using the MyCycler, Thermal Cycler (Bio-Rad, Laboratories, Inc., Hercules, CA) for 35 cycles, each at 95°C for 45 seconds, 58°C for 45 seconds, 72°C for 15 seconds, with a final extension at 72°C for 5 minutes. 1 µl of the UL30 amplified product was used for a second amplification of 35 cycles with the internal UL30 primers. Amplified products were detected by gel electrophoresis on 2% agarose gel, stained with ethidium bromide and visualized with UV light.

### SHIV-RT Plasma Load Detection

Plasma was separated from whole blood by centrifugation at 2000 rpm for 10 minutes, clarified at 2000 rpm for 10 minutes at 4°C and stored at −80°C in 1 ml aliquots. Virus loads were determined by quantitative RT-PCR assay for SIV gag RNA [37].

### Humoral Responses

Plasma samples obtained at the indicated times after infection were monitored for the presence of SIV envelope Abs by using an established ELISA protocol [38]. HSV-2 specific Abs were detected by HerpeSelect 2 ELISA IgG (Focus Diagnostic, Cypress, CA).

### Statistical Analyses

The Fisher's exact test was used for statistical comparison of the percentage of SHIV-RT infected animals in the different groups (GraphPad Prism version 5.02 for Windows, GraphPad Software, San Diego, CA). For all other pairwise comparisons, the semi-parametric Conover-Iman t-test on the ranked data was used (SAS 9.2 for Windows, The SAS Institute, Cary, NC). For the paired comparisons presented in Figure S3C, the Conover-Iman t-test on the sign-ranked data was used. P values<0.05 were taken as statistically significant.

## Results

### Long Term Shedding of HSV-2 DNA following Vaginal HSV-2 Challenge

**Figure 1:**
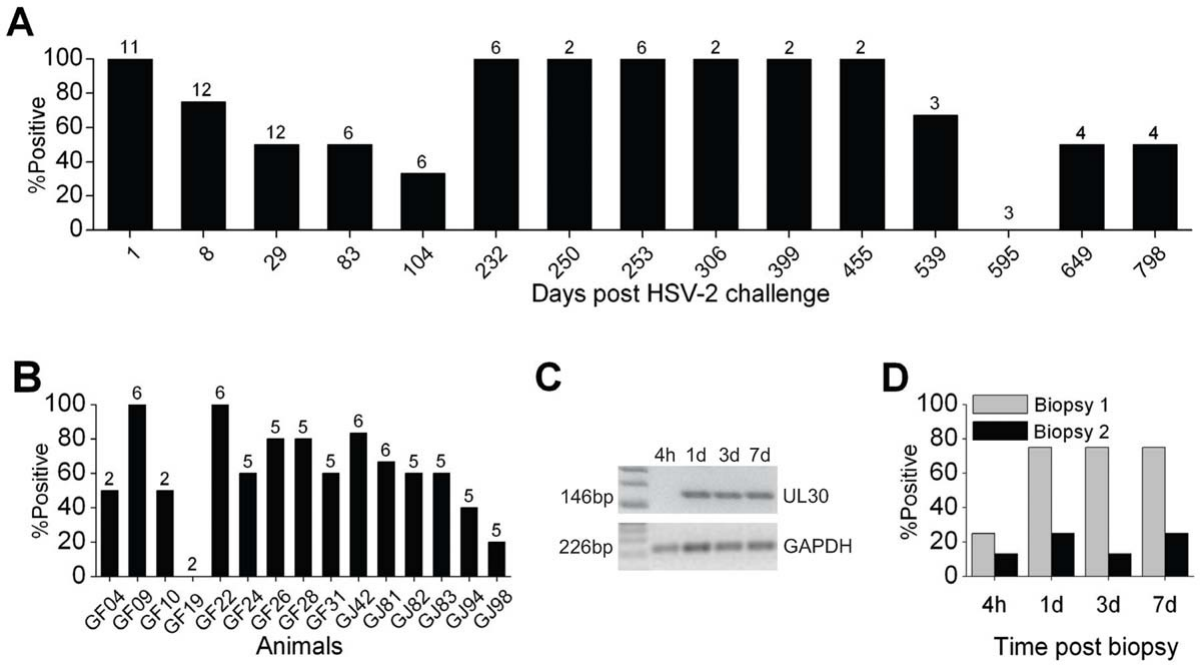
HSV-2 shedding after vaginal exposure. (A) Vaginal swabs were collected at the indicated times after HSV-2 exposure and HSV-2 DNA shedding was determined by measuring the presence of HSV-2 UL30 DNA by PCR. Each sample's integrity was verified by amplifying the GAPDH gene. The percentage of samples positive for HSV-2 UL30 DNA at the indicated time points is shown relative to the primary exposure to HSV-2. The numbers above the bars indicate the number of animals tested at each time point. (B) The DNA shedding is shown for each animal, where the numbers above the bars denote the number of times that animal was sampled during the study. (C,D) 3–6 (Biopsy 1) or 9–12 (Biopsy 2) months after co-challenge vaginal and cervical biopsies were taken from 8 animals and vaginal swabs were collected 4 hours, 1, 3 and 7 days later. (C) Representative gels resolving the amplified 146 bp HSV-2 UL30 gene and the 226 bp GAPDH gene control are shown for a set of samples from one animal. (D) The percentage of HSV-2 DNA positive animals for the indicated samples are provided (n = 8 for each biopsy).

To study the impact of HSV-2 on immunodeficiency virus infection, macaques were challenged with HSV-2 and later co-challenged with HSV-2 and SHIV-RT (Fig. S1). This approach was used as a proof of concept to maximize the chance of establishing HSV-2 infection and would model the human setting where someone first encounters HSV-2 and then is coincidently exposed to HSV-2 and HIV. Animals were Depo-Provera-treated in an attempt to render them more uniformly susceptible to HSV-2 (as seen for immunodeficiency virus infection [39]). Approximately 10% of the monkeys that were challenged i.vag. with HSV-2 developed cervical inflammation and/or lesions within 1 day of challenge and these resolved within 7–10 days (data not shown). Using a sensitive PCR assay to detect the HSV-2 UL30 gene (Fig. S2), long term HSV-2 DNA shedding into the vaginal fluids was demonstrated (Fig. 1). The input inoculum was detected in all animals tested 1 day after challenge, followed by varying levels of shedding over time (Fig. 1A; not all animals were tested at all time points). Although the available animal numbers were lower at the later time points, HSV-2 DNA was detected in 50% of the animals tested approximately 2 years after the primary HSV-2 challenge. More replicates of each sample (up to 6) were analyzed for the later time points in order to detect low level infections (e.g., Fig. S2). No HSV-2 DNA shedding was detected in one of the fifteen animals tested (GF19, only tested on two occasions), while the shedding frequency ranged from 20% to 100% in the remaining animals (Fig. 1B).

In an attempt to reactivate and increase HSV-2 DNA shedding as further proof of HSV-2 infection, cervical and vaginal pinch biopsies (2–4 each) were taken from 8 animals approximately 3–6 (Biopsy 1) and 9–12 (Biopsy 2) months after HSV-2/SHIV-RT co-challenge (once infected with SHIV-RT; approximately 15–24 months post primary HSV-2 exposure). The presence of HSV-2 DNA was monitored in the tissues (at biopsy) and in fluids (4 hours to 7 days post biopsy) and a representative PCR result for the fluids is shown in Figure 1C. After the first biopsy, HSV-2 DNA was detected in the cervical tissues of 1 of the animals but not in the vaginal tissues (data not shown), while HSV-2 DNA was detected in the swabs of 25% of the animals 4 hours later and in 75% of the animals within the next days (Fig. 1D). Considerably less shedding was seen after the second biopsy and, even after performing the PCR on multiple replicates of each sample (to increase our sensitivity of detection, Fig. S2), there was limited increased shedding at this late time point (Fig. 1D, only 10–20% of the animals showed any positivity by PCR). Thus, vaginal HSV-2 exposure of macaques resulted in long-term productive HSV-2 infection as evidenced by viral shedding for up to 2 years that progressively reduced.

### Vaginal HSV-2 Exposure Increases the Frequency of SHIV-RT Infection

**Figure 2:**
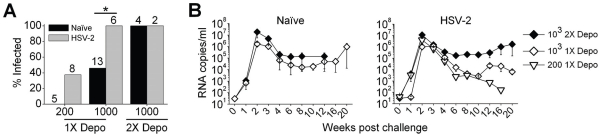
HSV-2-exposed macaques are more susceptible to vaginal SHIV-RT infection. (A) Naïve or HSV-2-exposed macaques were challenged with the indicated TCID_50_ of SHIV-RT or SHIV-RT and HSV-2 (respectively), after receiving one (1X) or two (2X) doses of Depo-Provera (Fig. S1) and the percentage of SHIV-RT-infected animals is shown (numbers above the bars indicate animal numbers per group). The asterisk marks the statistically significant (P<0.05) difference between 1X Depo-Provera-treated naïve vs HSV-2-infected animals receiving 10^3^ TCID_50_ of SHIV-RT. (B) Plasma viremia (mean RNA copies/ml±SEM) in SHIV-RT-infected naïve (left panel) and HSV-2-exposed (right panel) macaques post challenge with 10^3^ TCID_50_ after 2 doses of Depo (10^3^ 2X Depo - Naïve n = 4; HSV-2 n = 2) or after 1 dose of Depo (10^3^ 1X Depo - Naïve n = 6; HSV-2 n = 6) and 200 TCID_50_ after 1 dose of Depo (200 1X Depo - HSV-2 n = 3).

In order to determine if HSV-2-exposed macaques exhibited increased susceptibility to immunodeficiency virus infection (like humans), we co-challenged HSV-2-infected animals with HSV-2 and SHIV-RT. SHIV-RT, which expresses the reverse transcriptase of HIV in the context of SIV, was chosen as our challenge immunodeficiency virus since we intended to test the efficacy of a new generation NNRTI-containing microbicide gel in HSV-2-infected animals, which has been shown to be effective in HSV-2 naïve animals [28]. The initial SHIV-RT challenges (10^3^ TCID_50_) revealed that all HSV-2-exposed and naïve animals that received two Depo-Provera treatments within 11 weeks prior to SHIV-RT challenge became infected (Fig. 2A, 2X Depo). In these animals, the second Depo-Provera treatment was 6 weeks after vaginal HSV-2 challenge. This contrasts with the ∼46% SHIV-RT infection frequency seen in 1X Depo-Provera-treated naïve animals [28]. Consequently, it was impossible to elucidate if HSV-2 infection impacted the transmission frequency of SHIV-RT. Although Depo-Provera thins the cervicovaginal epithelium to increase permissivity to viral infection to reproducible levels, it is also immunosuppressive [40], [41]. This is likely exacerbated when 2 doses are given within a relatively short 3 month period. Thus, additional animals were Depo-Provera treated 4–7 months after the initial vaginal HSV-2 challenge, before being co-challenged (Fig. S1). In addition, animals that had been originally challenged with HSV-2 via the rectal route (and were, therefore, not previously Depo-Provera-treated) were Depo-Provera-treated and co-challenged with HSV-2 and SHIV-RT (Fig. S1). Such HSV-2-challenged animals receiving only a single Depo-Provera treatment in the 3 month period exhibited significantly increased susceptibility to SHIV-RT infection compared to naïve animals (Fig. 2A, 1X Depo); 100% SHIV-RT infection in the HSV-2 group (6 of 6 animals) vs ∼46% SHIV-RT infection in the naïve group (6 of 13 animals), when challenged with 10^3^ TCID_50_ of SHIV-RT (Fig. 2A) (P<0.05). This was similar in animals challenged with the lower 200 TCID_50_ dose of SHIV-RT (Fig. 2A, 1X Depo); ∼37% SHIV-RT infection in the HSV-2 group (3 of 8 animals) vs 0% SHIV-RT infection in the naïve group (0 of 5 animals; not statistically significant). Despite the increased frequency of infection in the 1X Depo-Provera-treated HSV-2 exposed animals, comparable levels and kinetics of plasma viremia were observed in all infected animals, with peak levels occurring typically 2 weeks after challenge (Fig. 2B). Interestingly, infected animals that were treated twice, vs single treatment with Depo-Provera, maintained higher SHIV-RT viremic set-points for 6 months post-infection, although peak viral loads were comparable (Fig. 2B).

### HSV-2 Exposure Elicits Acute Innate and Long Lasting Adaptive Responses

**Figure 3:**
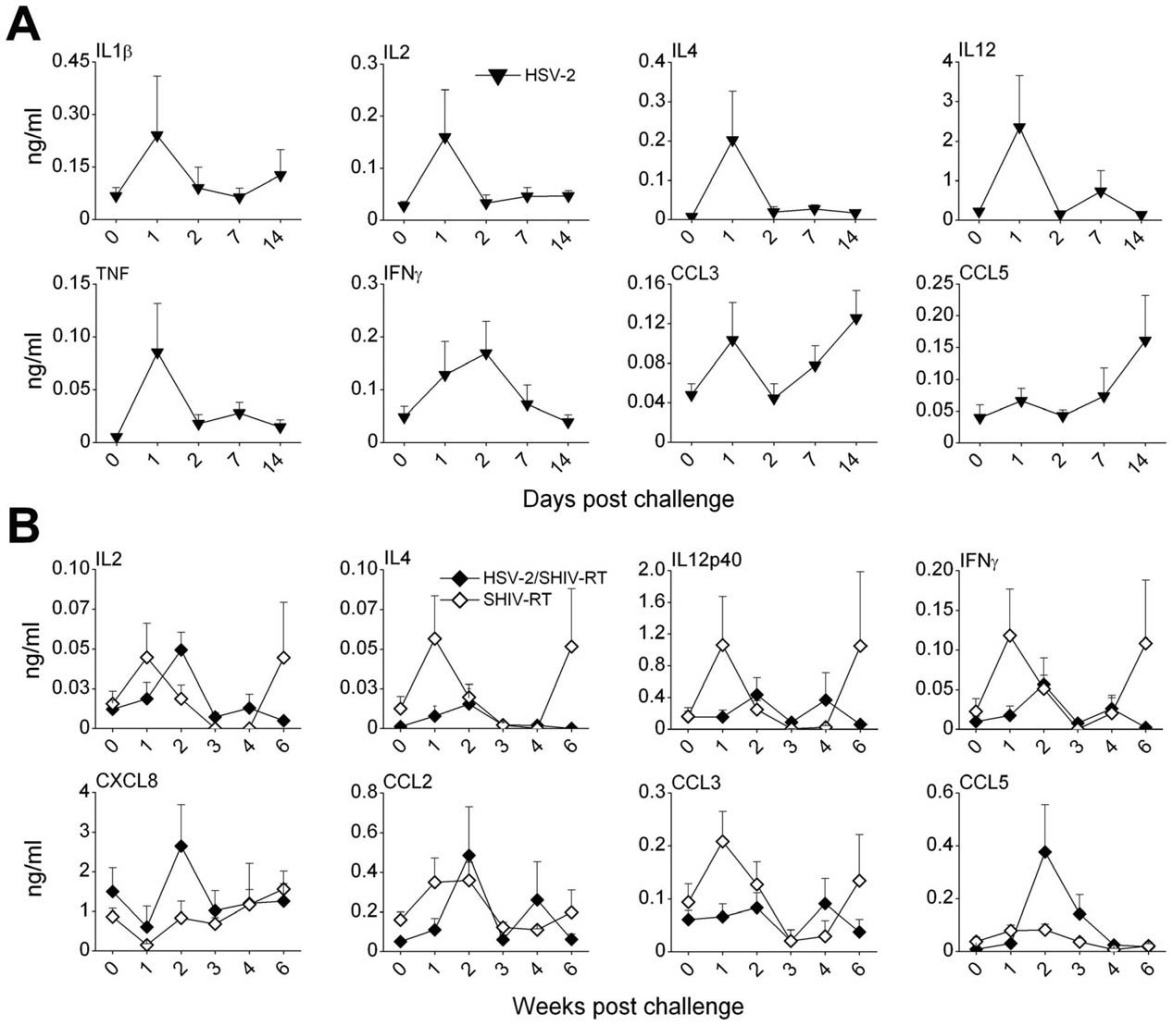
Local immune responses following HSV-2 infection. (A) Naïve Depo-Provera-treated monkeys (n = 12) were challenged i.vag. with 2×10^8^ pfu HSV-2. Cytokine/chemokine levels were measured in the vaginal swabs during the first days after challenge using the 14-plex Luminex assay. The mean concentration ng/ml (±SEM) of each factor is shown. (B) Vaginal swabs were collected after HSV-2/SHIV-RT co-challenge of HSV-2-infected animals (n = 5, closed symbols) or SHIV-RT challenge of naïve animals (n = 6, open symbols). The cytokine/chemokine levels (mean±SEM ng/ml) were measured by Luminex assay on a weekly basis.

During the first days of primary HSV-2 exposure, acute responses to HSV-2 were observed in the vaginal swabs of all monkeys (Fig. 3A). Production of IL1β, IL2, IL4, IL12, IFNγ, TNFα, and CCL3 peaked 1–2 days post challenge, while CCL3 and CCL5 levels increased again around 14 days after challenge. CXCL8 levels initially decreased going back to pre-challenge levels within 14 days (data not shown).

Local cytokine and chemokine responses were further monitored over the first 6 weeks of HSV-2/SHIV-RT co-challenge and compared to the responses seen in naïve animals infected solely with SHIV-RT. Elevated cytokine (IL2, IL4, IL12p40, and IFNγ) and chemokine (CCL2, CCL3, and CCL5) levels were detected within 1 week of SHIV-RT infection of naïve macaques, but these were often lower or delayed in the HSV-2/SHIV-RT co-infected animals (Fig. 3B; although none of these differences reached statistical significance. Note the smaller scales for the cytokines in panels A and B.) Levels tended to normalize to baseline values within 3–4 weeks of infection in all animals, with IL2, IL4, IL12p40, IFNγ, and CCL3 increasing again after 6 weeks of SHIV-RT infection of the naïve animals (not statistically significant). After the initial decrease in CXCL8 levels in both groups of animals, the levels normalized to pre-infection levels.

**Figure 4:**
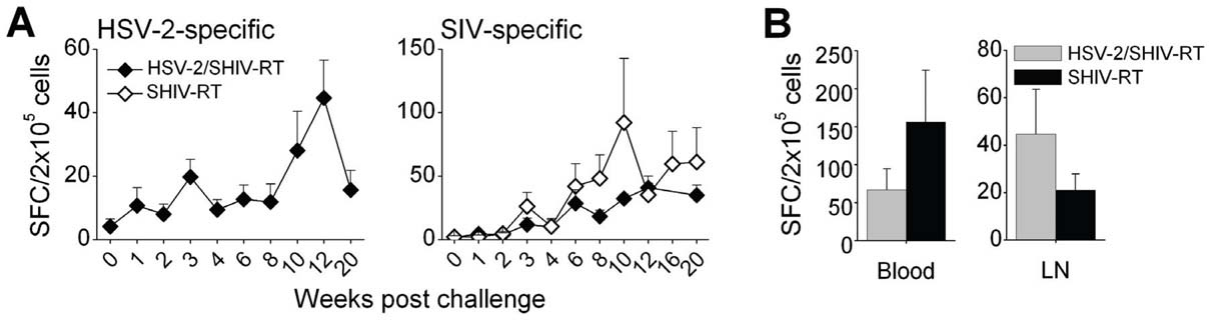
HSV-2 infection induces adaptive immunity and does not impede SIV-specific adaptive responses. (A) HSV-2- and SIV-specific T-cell responses were monitored over time by IFN-γ ELISPOT and the data are expressed as the mean (±SEM) HSV-2- (left panel) or SIV-specific (right panel) spot-forming cells (SFC) per 2×10^5^ PBMCs. (HSV-2/SHIV-RT co-infected closed symbols, n = 8; SHIV-RT-infected open symbols, n = 10). (B) The mean (±SEM) numbers of SIV-specific SFC per 2×10^5^ cells are plotted for the blood (left panel) and for the LNs (right panel) collected upon euthanization of HSV-2/SHIV-RT co-infected animals (n = 8, 10–13 months post co-challenge) vs animals only infected with SHIV-RT (n = 3, 10–12 months post challenge).

The ELISPOT assay, measuring IFNγ-secreting antigen-specific T cell responses, was used to assess adaptive cellular responses to both HSV-2 and SHIV-RT infection over time. HSV-2 specific cellular responses were not seen prior to HSV-2 exposure (<2 SFC/2×10^5^ cells, averaged from 3 baseline bleeds prior to challenge; not shown). Minimal HSV-2-specific responses were seen after primary HSV-2 challenge in the short time prior to co-exposure to HSV-2 and SHIV-RT (sampled on 1, 2, 3, 4, 6, and 11 weeks post challenge; week 11 post primary HSV-2 challenge is the week 0 in Fig. 4A, left panel). After co-challenge these responses increased (Fig. 4A, left panel; HSV-2-specific responses were not monitored in SHIV-RT-infected animals). Comparable numbers of SIV-specific IFNγ-producing cells were found in the PBMCs of SHIV-RT-infected and HSV-2/SHIV-RT co-infected animals (Fig. 4A, right panel). SIV-specific T cell responses persisted over time in both groups, with comparable responses being detected in the blood and lymph nodes (∼10 months post SHIV-RT challenge; Fig. 4B).

To evaluate if depletion of CD8^+^ lymphocytes would affect HSV-2-specific viral and immune parameters in HSV-2/SHIV-RT co-infected macaques, we depleted HSV-2/SHIV-RT co-infected and SHIV-RT-infected (∼6 months after SHIV-RT exposure for both groups) monkeys of CD8^+^ cells [29].

CD8^+^ T cells were depleted from the peripheral blood within 3 days of Ab administration and started to rebound at day 21 (Fig. S3A). In all animals the plasma SHIV-RT levels increased within 3–7 days of anti-CD8 mAb treatment (21±7.1 and 16±14.2 mean fold increase at day 3 and 34.3±14 and 11±8.1 mean fold increase at day 7 for HSV-2/SHIV-RT co-infected and SHIV-RT-infected animals, respectively) and returned to pre-depletion levels when the levels of CD8^+^ cells began to normalize (Fig. S3B). HSV-2 DNA shedding was not enhanced during CD8^+^ cell depletion and in fact tended to decrease over time, with an average of 35.7% shedding in the 8 weeks following the treatment (each of the 7 animals was positive at least once in the 10 samples taken per animal over this period), without any obvious lesions developing. Since HSV-2 and HIV/SIV are known to affect DC biology [35], [42]–[44], CD80 and CD86 levels were monitored on blood PDCs (Lineage^-^HLA-DR^+^CD123^+^ cells) and MDCs (within the CD123^−^ fraction for the Lineage^−^HLA-DR^+^ cells) [33], [35] during CD8^+^ cell depletion (Fig. S3C). Starting levels of CD80/CD86 expression were comparable between the groups. CD80/CD86 expression increased during CD8^+^ cell depletion (days 7–14), but this only reached statistical significance (peak levels relative to the pre-treatment baseline levels) for CD80 expression by MDCs in co-infected animals after 7 days (P<0.002, Fig. S3C, lower left panel), and for CD80 expression by PDCs in co-infected animals after 14 days (P<0.002) (Fig. S3C, lower right panel). CD80/CD86 expression decreased from peak between days 14 and 21 of treatment (P<0.002 for CD80/CD86 expression on both MDCs and PDCs in co-infected animals; peak vs day 21 values). Although the trends were similar, no significant differences were observed in CD80/CD86 expression in SHIV-RT-infected animals, likely due to the limited number of animals in this group. CD80/CD86 expression began increasing towards baseline levels from day 42. At day 7 of treatment, the elevated CD80 expression by PDCs (Fig. S3C, lower right panel) in SHIV-RT-infected animals was significantly greater than that in co-infected macaques (P<0.03, Fig. S3C, lower right panel). In addition, HSV-2/SHIV-RT co-infected animals exhibited an approximately one-week delay in rebound toward baseline levels. Coincident with this delay, at day 42, CD80 and CD86 expression on MDCs, and CD86 on PDCs were significantly lower in co-infected vs SHIV-RT-infected animals (P<0.05). MDC and PDC percentages did not change after CD8^+^ cell depletion (data not shown). HSV-2- and SIV-specific IFNγ responses by PBMCs decreased during CD8^+^ cell depletion (Fig. S3D, peak effect after 7 days). HSV-2-specific responses were reduced ∼2 fold in the HSV-2/SHIV-RT co-infected animals at day 7 after CD8^+^ cell depletion (Fig. S3D, left panel). While SIV-specific IFNγ responses decreased ∼2 fold in the SHIV-RT-infected animals, there was a ∼9 fold reduction in HSV-2/SHIV-RT co-infected animals at day 7 (Fig. S3D, right panel). However, the difference between the two groups did not reach statistical significance.

**Figure 5:**
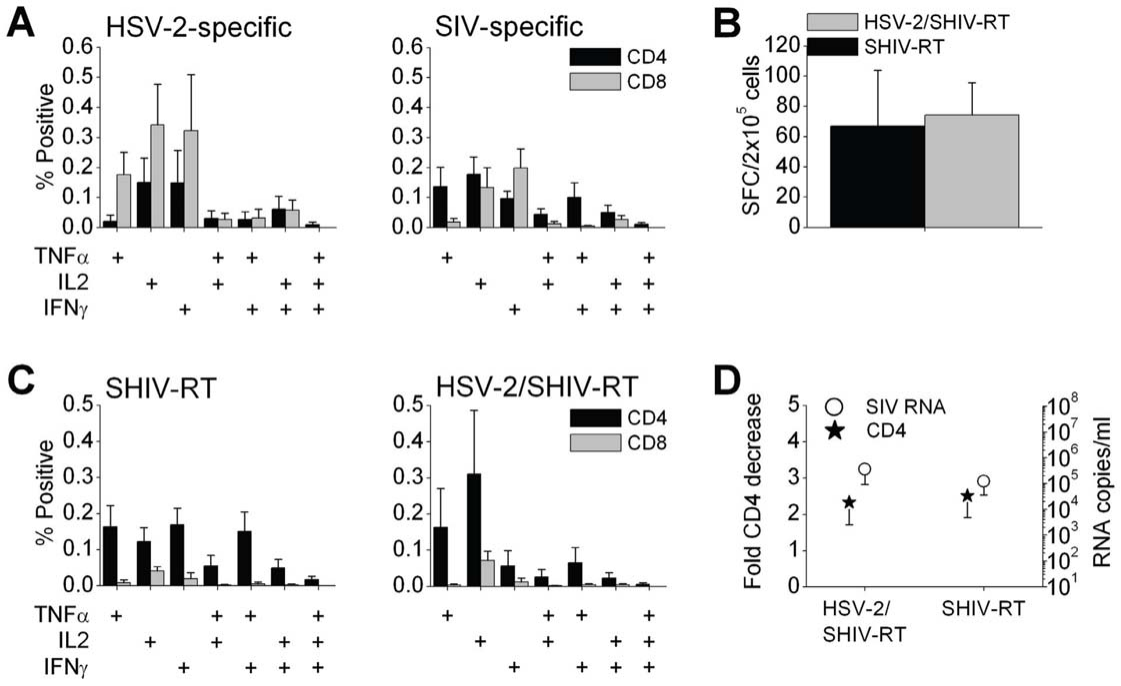
HSV-2-specific CD4^+^ and CD8^+^ T cell responses are detected in HSV-2/SHIV-RT co-infected animals. (A) HSV-2- (left panel) and SIV-specific (right panel) CD4^+^ (black bars) and CD8^+^ (grey bars) T cells were detected in the blood of co-infected animals (∼12 months after co-challenge, n = 8) by multicolor ICS. The percent positive cells producing the indicated cytokines in response to each antigen (mean±SEM; single vs double or triple producers) are shown after subtracting the respective background signals of the IgG and no-virus MV controls. (B,C) SIV-specific T-cell responses were compared between SHIV-RT-infected (n = 5) and HSV-2/SHIV-RT co-infected (n = 5) animals ∼12 months post co-challenge, by IFNγ ELISPOT (B) or ICS (C). (B) The mean (±SEM) SIV-specific SFC/2×10^5^ cells are shown. (C) The percent positive cells producing the indicated cytokines in response to SIV (mean±SEM; single vs double or triple producers) are shown after subtracting the respective background signals of the IgG and no-virus MV control. (D) Approximately 12 months after challenge with SHIV-RT or co-challenge with HSV-2/SHIV-RT, the fold decreases in CD4 counts (mean±SEM, relative to pre-exposure levels) (left axis, stars) and plasma viral loads (mean RNA copies/ml±SEM, right axis, circles) are shown (n = 5 for both).

Antigen-specific T cell responses were examined in more detail by ICS to more accurately dissect the involvement of CD4^+^ and CD8^+^ T cells (∼12 months post SHIV-RT or HSV-2/SHIV-RT challenge). In HSV-2/SHIV-RT co-infected animals both HSV-2- and SIV-specific CD4^+^ and CD8^+^ T cells were found to produce IFNγ, IL2, or TNFα alone, with relatively fewer cells typically producing two or more of these cytokines (Fig. 5A). HSV-2-specific CD8^+^ T cell responses were typically stronger than the CD4^+^ T cell responses, but this did not reach statistical significance. To determine whether HSV-2 infection affected SIV-specific T cell responses in SHIV-RT-infected animals, we compared PBMCs from both groups using ELISPOT and ICS. By ELISPOT assay, SIV-specific responses were similar in SHIV-RT-infected and HSV-2/SHIV-RT-co-infected animals (Fig. 5B), as seen at the earlier time points (Fig. 4A). Additionally, there was no significant difference in the SIV-specific cytokine secretion profiles from both CD4^+^ and CD8^+^ T cells between SHIV-RT-infected vs co-infected animals (Fig. 5C). Within each infection group, CD4^+^ T cells predominantly expressed cytokines, while the CD8^+^ T cell response was lower than 0.05% (at this time point; Fig.5C). Taken together, these results are consistent with there being no difference in the decrease in CD4 counts (relative to pre-infection) and the levels of plasma SHIV-RT RNA in HSV-2/SHIV-RT co-infected vs SHIV-RT infected animals at this time point (Fig. 5D).

SIV-specific Ab responses were detected in all SHIV-RT-infected and in 85% of the HSV-2/SHIV-RT co-infected animals (Tables 1 and 2). Low-level HSV-2-specific Ab responses were detected in 75% of the HSV-2/SHIV-RT co-infected animals (mean 2.32±0.54 fold increase in OD above the pre-challenge values in 9 of 12 animals tested). Two of the HSV-2 Ab negative animals were positive for HSV-2 DNA shedding (GF24 and GF22), while animal GF19 was both Ab and HSV-2 DNA negative.

### PC-817 Protects against Enhanced Vaginal SHIV-RT Infection in HSV-2-Exposed Macaques

**Figure 6:**
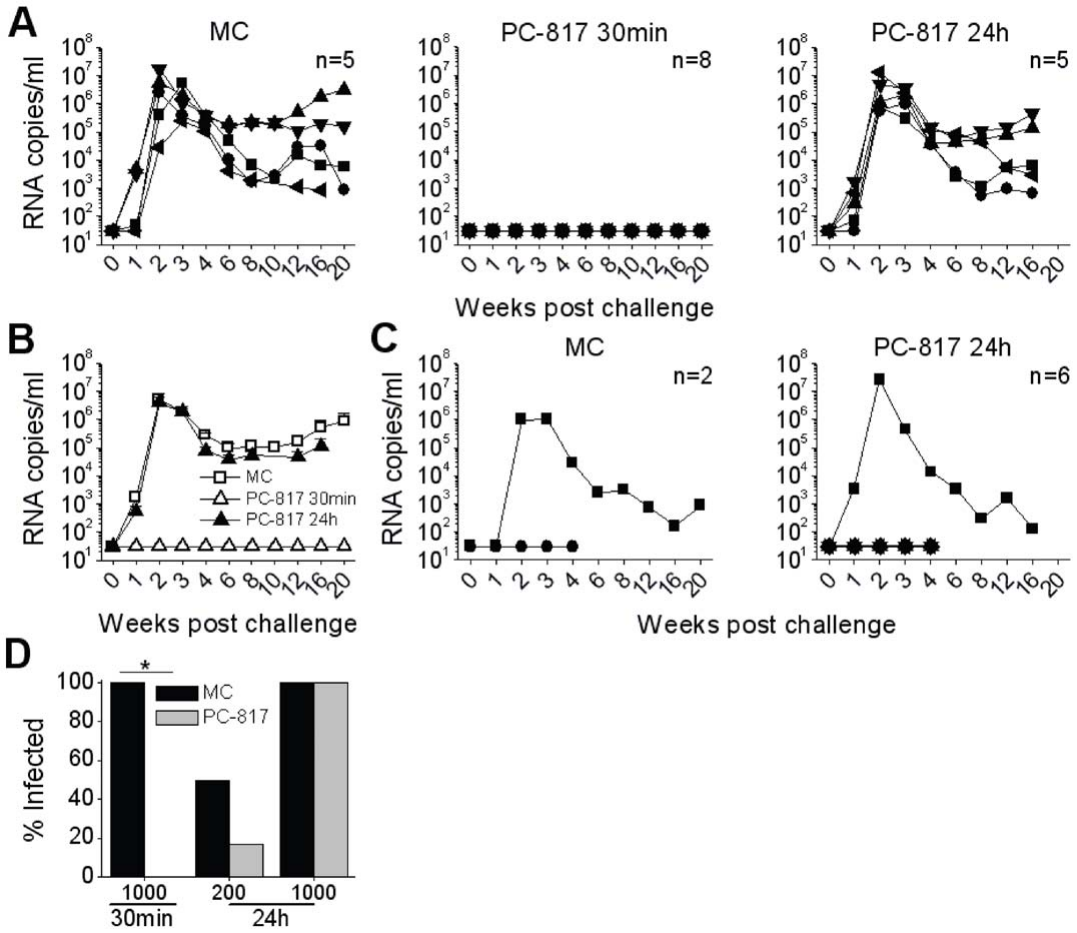
PC-817 protects against vaginal SHIV-RT infection when given just prior to HSV-2/SHIV-RT co-challenge. (A) PC-817 or MC was applied 30 minutes (30 min) and 24 hours (24 h) prior to challenge of HSV-2-exposed animals with 10^3^ TCID_50_ SHIV-RT and 2×10^8^ pfu HSV-2. Plasma viral loads are shown for control animals (MC) (n = 5) in the left panel, for animals that received PC-817 30 min before the challenge (n = 8) in the middle panel, and for animals that received PC-817 24 h prior to challenge (n = 5) in the right panel. (B) Mean (±SEM) plasma viremia of the 3 treatment groups from (A) are shown. (C) PC-817 or MC was applied 24 h prior to challenge with 200 TCID_50_ of SHIV-RT and 2×10^8^ pfu HSV-2. The plasma viral loads are provided for the MC control animals (n = 2) in the left panel and the PC-817-treated animals (n = 6) in the right panel. (D) Data from panels (A) and (C) are summarized to show the frequency of infection for the differently treated groups. The asterisk highlights the statistically significant (P<0.001) difference in the numbers of infected animals receiving PC-817 (vs MC) 30 min prior to challenge with 10^3^ TCID_50_ of SHIV-RT and HSV-2.

Having demonstrated HSV-2 infection of macaques and that HSV-2 exposure significantly enhanced the frequency of SHIV-RT infection in this model, we wanted to utilize this system to carry out the first test of a lead microbicide gel comprising the NNRTI MIV-150 in Carraguard (PC-817) for its ability to prevent SHIV-RT infection under these more rigorous conditions. PC-817 was tested for its ability to prevent vaginal SHIV-RT infection when applied 30 minutes or 24 hours prior to co-challenge of HSV-2-exposed animals with HSV-2 and SHIV-RT (Fig. 6) vs a placebo control gel, MC.

Just as in naïve animals challenged with SHIV-RT [28] (unpublished observations), PC-817 administered 30 minutes prior to exposure completely prevented SHIV-RT infection in HSV-2 infected macaques (Fig. 6A, middle panel) despite their enhanced susceptibility to infection (Fig. 2). However, PC-817 no longer protected when applied 24 hours before the 10^3^ TCID_50_ inoculum (Fig. 6A, right panel). The mean viral loads of the infected animals were comparable in the animals treated with MC or pretreated with PC-817 24 hours before challenge (Fig. 6B). Pre-treatment with PC-817 24 hours before challenge appeared partially effective when the HSV-2-infected animals were co-challenged with the lower 200 TCID_50_ inoculum of SHIV-RT and HSV-2 (Fig. 6C, 50% infection in MC vs 16.7% infection in PC-817-treated animals; not statistically significant, likely due to the small number of animals in the MC group). As expected, all plasma viremia positive animals carried infectious virus in their blood cells and most developed both SIV-specific IFNγ-producing cells and Abs (Table 2). The percentage of infected animals in each of the treatment groups is summarized in Figure 6 D. The potent effect of PC-817, when administered just prior to exposure, in preventing SHIV-RT infection in HSV-2-infected animals despite their enhanced susceptibility to SHIV-RT infection is clear (P<0.001).

## Discussion

HSV-2 enhances HIV acquisition and transmission during symptomatic and asymptomatic stages of infection [3], [4], [18]. The exact mechanism via which HSV-2 does this remains unknown, due to the difficulties in studying human infections and the lack of an animal model to study HSV-2/immunodeficiency virus co-infection [45], [46]. However, recent studies have revealed that, even after anti-HSV-2 therapy, recruited HIV receptor-positive T cells and DCs persist at sites of HSV-2 reaction, thereby possibly contributing to continued HIV replication and spread [21]. Herein, we describe the first relevant primate HSV-2/SHIV-RT co-infection model that can be used to closely dissect the mechanism by which HSV-2 enhances immunodeficiency virus spread. Moreover, this model allows the assessment of promising anti-viral strategies for their ability to prevent immunodeficiency virus infection under these more physiologically demanding conditions. The rationale for first exposing animals to HSV-2 and then co-challenging them with HSV-2 and SHIV-RT was to give us the best chance of getting HSV-2 infection (with the limited numbers of animals available for this study), since this is something that has not documented previously. While HSV-2 infected humans might be more likely exposed to HIV alone, it is not impossible that someone infected with HIV and HSV-2 could spread both viruses (or at least HIV) to another individual (who might already by HSV-2 positive) during one encounter. This study was carried out to provide the first proof that HSV-2 can infect macaques vaginally and that HSV-2 exposure enhances SHIV-RT infection. Future studies are now justified to explore sequential exposure vs co-exposure with both viruses, in order to further dissect how HSV-2 alters the susceptibility to HIV infection. This work provides the first important step towards that goal.

Although HSV-2 infection of humans can be asymptomatic [18], [47], [48], viral shedding occurs frequently, with the median duration of a shedding episode estimated to be between 6–48 h [8], [49]. More recent evidence further supported that shedding and lesions are rapidly cleared and typically asymptomatic [12]. We observed frequent shedding in HSV-2-challenged macaques for approximately 2 years, but this might even be an underestimate if the short window of shedding was missed in our sampling schedules. Increased HSV-2 shedding was detected after biopsying the cervical and vaginal tissues (especially earlier in infection), despite there being little evidence of DNA positivity at the time of biopsy, suggesting that the trauma associated with the biopsies reactivated shedding. HSV-2 detection was reportedly higher in recently infected compared to chronically infected people [50]. Since all HSV-2-challenged animals were ultimately co-challenged with HSV-2 and SHIV-RT we were unable to accurately determine if HSV-2 shedding significantly decreased over time in macaques, although there was less reactivation upon biopsying at the later time points.

HSV-2 vaginal challenge in macaques induced acute local cytokine and chemokine responses, as well as adaptive T and B cell responses. Because we were unable to include controls of animals exposed to UV-inactivated HSV-2, we cannot rule out that the local innate responses were not simply due to a ligand effect of cells responding to the virus particles. However, in mice vaginally challenged with HSV-2, early production of chemokines such as CCL2 and CCL5 in vaginal mucosa has been reported [51], as well as massive recruitment of B cells from the vaginal mucosa to the lymph nodes [52]. Type I IFNs, β-chemokines, IL1, IL6 and IL12 are also released in human herpetic lesions [7]. Similar cytokine and chemokine responses detected in the first days after vaginal HSV-2 exposure of macaques likely contribute to the recruitment of T cells, macrophages, and DCs that are important in controlling infection [8], [9], [21]. The somewhat delayed local IL2 and CCL5 and lower CCL3 responses observed after co-challenge of HSV-2-infected macaques compared to those detected after SHIV-RT infection of naïve animals, suggest that HSV-2 impaired the responses to SHIV-RT. Not surprisingly, this did not ultimately inhibit the development of SIV-specific T or B cell responses, since these typically develop in most immunodeficiency virus infected animals. This contrasts with a recent report that co-infection with HSV-2 and HIV was associated with a weaker HIV-specific T cell responses [53].

CD8^+^ T cells play a pivotal role in controlling HSV-2 infection and reactivation locally in the peripheral mucosa [8], [21] and in controlling SIV infection [54]. As expected, SIV RNA levels increased and antigen-specific T cell responses decreased upon CD8^+^ cell depletion, indicating the involvement of CD8^+^ cells in the HSV-2- and SIV-specific responses of macaques. HSV-2-specific CD4^+^ and CD8^+^ T cells producing TNFα, IL2, or IFN γ were also identified in the blood of co-infected animals by ICS, with the CD8^+^ T cell responses being greater. T cells producing combinations of two or all of these factors were less frequent. Despite the depletion of CD8^+^ cells, there was no increase in HSV-2 shedding in the vaginal swabs. This might be explained by sub-optimal CD8^+^ cell depletion in genital tissue, since we did not monitor CD8^+^ cells locally.

The changes in CD80/CD86 expression by DCs during CD8^+^ cell depletion were similar in SHIV-RT-infected and HSV-2/SHIV-RT co-infected animals, suggesting that the DC activation was likely due to SHIV-RT-driven activation when the plasma virus loads increased with CD8^+^ cell depletion. SIV and HIV activate PDCs, with at least some bystander activation of MDCs [33], [42], but this activation is often suboptimal for full immune stimulation [35]. The rebound of CD80/CD86 expression was delayed in the HSV-2/SHIV-RT co-infected animals, although this was only evident at one time point. We showed previously that HSV-2-infected monocyte-derived DCs were defective in their ability to stimulate SIV-specific T cell responses *in vitro* [17]. Reduced CD83 expression by HSV-2-exposed DCs has been associated with lessened immunostimulatory function [16], [55], [56], but CD83 was not monitored in our study. However, we did not observe lower SIV-specific responses in the blood of the co-infected animals, which might have been more apparent at local infection sites or sites of reactivation where DCs would be more likely to become infected by HSV-2.

HSV-2 exposure increased the monkeys' susceptibility to vaginal SHIV-RT infection, supporting the observations of increased HIV infection in HSV-2 seropositive people, with or without detectable herpetic lesions [3], [4]. Lesions were not evident upon HSV-2/SHIV-RT co-challenge. A possible ligand effect of HSV-2 exposure in the co-challenge cannot be excluded based on the way these experiments were performed. While we cannot rule out the contribution of recruited DCs and T cells to microlesions not visible by colposcopy [21], it is possible that the immunosuppressive activities of HSV-2 infection also contribute to the animals' increase susceptibility to SHIV-RT infection. Depo-Provera not only thins the vaginal epithelium rendering animals more uniformly susceptible to vaginal challenge [40], it has also been shown to immunosuppress animals, undermining the protective capacity of nef-defective SIV [41]. The fact that the double Depo-Provera treatment (within a 3 month period) somewhat mirrored the HSV-2 effect in naïve animals, supports the idea that immunosuppression can contribute to immunodeficiency virus infection. Despite the increased frequency of immunodeficiency infection in HSV-2-infected macaques, we did not observe any significant changes in the SHIV-RT plasma viral loads, as has been described in humans (0.33–1 log more HIV in HSV-2-infected people) [2].

Using this HSV-2/SHIV-RT model in which the SHIV-RT infection frequency is enhanced, we tested whether a promising new generation microbicide (using Carraguard as the delivery vehicle) was still able to protect against SHIV-RT infection as it did in naïve animals [28]. Although Carraguard did not demonstrate efficacy against HIV infection in women in a recent efficacy study, it was found to be safe and acceptable [57]. As such, new generation formulations are being explored, where Carraguard serves as the vehicle to introduce other anti-viral components. Herein, we tested Carraguard combined with MIV-150 (a combination formulation designated PC-817); *in vitro* the two components have been shown to have additive activities against HIV [28], [58]. Just as we observed in naïve macaques [28] (unpublished observations), PC-817 completely prevented SHIV-RT infection when applied just prior to challenge, but exhibited little or no activity when applied 24 hours prior to challenge. This has important implications for the advancement of coitally independent strategies that function in naïve and HSV-2-infected settings, and studies examining different timing strategies, as well as repeated gel applications are ongoing.

This represents the first report demonstrating vaginal HSV-2 infection of non-human primates and that macaques exposed to HSV-2 are more susceptible to immunodeficiency virus infection, thereby mimicking human biology. We also verified how this model could be used to test promising microbicide candidates. We established an animal model that could be critical to elucidate how HSV-2 facilitates HIV spread and to more rigorously test the efficacy of novel preventative and/or therapeutic approaches in the presence of other sexually transmitted pathogens.

## Supporting Information

Figure S1 Treatment and challenge regimens. Schematic diagram to detail the Depo-Provera treatments (1X vs 2X within 3 months) and challenge schedules of macaques vaginally or rectally exposed to HSV-2 prior to HSV-2/SHIV-RT co-challenge, vs naïve animals challenged with SHIV-RT.
(1.04 MB EPS)

Figure S2 PCR detection of low levels of HSV-2 DNA. 2×10^6^ pfu of HSV-2 was used to infect T75 flasks confluent with Vero cells (∼8.4×10^6^ cells). Infected Vero cells were collected 48 hours post infection (when 100% cells were rounded up from the bottom of the culture flask). Aliquots containing 5×10^6^ HSV-2-infected or uninfected Vero cells were prepared. Cell suspensions were centrifuged at 1500 rpm for 5 minutes at room temperature, the supernatant removed, and dry cell pellets stored at -80°C until DNA extraction. DNA was extracted using the QIAamp DNA blood Mini Kit (QIAGEN, Valencia, CA) according to manufacturer's instructions, into 100 µl of elution buffer. DNA from 10-fold dilutions of HSV-2 infected Vero cells (5 down to 0.00005 infected cells) was diluted on a background of DNA from 45,000 uninfected Vero cells. HSV-2 UL30 DNA was amplified by nested PCR on 6 replicates for each dilution. GAPDH signals were detected in all samples (not shown). Gels representative of at least 3 repeat experiments are shown.
(0.91 MB EPS)

Figure S3 Impact of CD8^+^ cell depletion on HSV-2/SHIV-RT and SHIV-RT-infected animals. Blood was sampled before, during, and after CD8^+^ cell depletion of 2 SHIV-RT-infected (open symbols) and 7 HSV-2/SHIV-RT co-infected (closed symbols) animals; CD8^+^ cell depletion was initiated ∼6 months post co-challenge with HSV-2/SHIV-RT or challenge with SHIV-RT. Results are shown relative to the days post treatment with the anti-CD8 mAb. (A) The CD8^+^ T cell TruCount results (numbers per µl of blood) are shown for the individual animals over time relative to the commencement of the Ab treatment. (B) The levels of SIV gag RNA were measured over time and the RNA copies/ml in plasma are shown for each animal. (C) Co-stimulatory molecule expression was measured on CD123^−^ MDCs and CD123^+^ PDCs within the Lineage^−^HLA-DR^+^ cell population obtained from PBMCs. Mean fluorescence intensities (MFIs, ±SEM) of CD86 (upper panels) and CD80 (lower panels) expression by CD123^−^ (CD123-) and CD123^+^ (CD123+) cells are shown over time. Asterisks indicate statistically significant differences between the HSV-2/SHIV-RT co-infected vs SHIV-RT-infected animals at specific time points. (D) Since the magnitudes of the responses varied significantly between animals, the fold reduction in the HSV-2- and SIV-specific IFNγ release was calculated by dividing the mean numbers of SFCs at day 0 by the mean numbers of SFCs at day 7 or day 14 of anti-CD8 Ab treatment for the respective responses. The mean fold reductions (±SEM) are shown for 7 HSV-2/SHIV-RT co-infected and 2 SHIV-RT-infected animals. Negligible HSV-specific responses in SHIV-RT-infected animals remained unchanged as expected (fold ∼1).
(3.26 MB EPS)
